# Antiviral Mechanisms of Saucerneol from *Saururus chinensis* against Enterovirus A71, Coxsackievirus A16, and Coxsackievirus B3: Role of Mitochondrial ROS and the STING/TKB-1/IRF3 Pathway

**DOI:** 10.3390/v16010016

**Published:** 2023-12-21

**Authors:** Jae-Hyoung Song, Seo-Hyeon Mun, Heejung Yang, Yong Soo Kwon, Seong-Ryeol Kim, Min-young Song, Youngwook Ham, Hwa-Jung Choi, Won-Jin Baek, Sungchan Cho, Hyun-Jeong Ko

**Affiliations:** 1 Department of Pharmacy, Kangwon National University, Chuncheon 24341, Republic of Korea; thdwohud@naver.com (J.-H.S.); moonnari0606@gmail.com (S.-H.M.); heejyang@kangwon.ac.kr (H.Y.); alsdudthd2097@naver.com (M.-y.S.); 2 Kangwon Institute of Inclusive Technology, Kangwon National University, Chuncheon 24341, Republic of Korea; 3 Division of Acute Viral Diseases, Centers for Emerging Virus Research, National Institute of Health, Korea Disease Control and Prevention Agency, Cheongju 28159, Republic of Korea; ksr12134@nate.com; 4 Nucleic Acid Therapeutics Research Center, Korea Research Institute of Bioscience and Biotechnology (KRIBB), Cheongju 28116, Republic of Korea; hyt5272@kribb.re.kr; 5 Department of Biomolecular Science, KRIBB School of Bioscience, Korea University of Science and Technology (KUST), Daejeon 34113, Republic of Korea; 6 Department of Beauty Art, Youngsan University, 142 Bansong Beltway, Busan 48015, Republic of Korea; rerived@naver.com (H.-J.C.); vdnjswls@naver.com (W.-J.B.)

**Keywords:** enterovirus A71, coxsackievirus A16, saucerneol, mitochondrial ROS, antiviral

## Abstract

Enterovirus A71 (EV71), coxsackievirus A16 (CVA16), and coxsackievirus B3 (CVB3) are pathogenic members of the *Picornaviridae* family that cause a range of diseases, including severe central nervous system complications, myocarditis, and pancreatitis. Despite the considerable public health impact of these viruses, no approved antiviral treatments are currently available. In the present study, we confirmed the potential of saucerneol, a compound derived from *Saururus chinensis*, as an antiviral agent against EV71, CVA16, and CVB3. In the in vivo model, saucerneol effectively suppressed CVB3 replication in the pancreas and alleviated virus-induced pancreatitis. The antiviral activity of saucerneol is associated with increased mitochondrial ROS (mROS) production. In vitro inhibition of mROS generation diminishes the antiviral efficacy of saucerneol. Moreover, saucerneol treatment enhanced the phosphorylation of STING, TBK-1, and IRF3 in EV71- and CVA16-infected cells, indicating that its antiviral effects were mediated through the STING/TBK-1/IRF3 antiviral pathway, which was activated by increased mROS production. Saucerneol is a promising natural antiviral agent against EV71, CVA16, and CVB3 and has potential against virus-induced pancreatitis and myocarditis. Further studies are required to assess its safety and efficacy, which is essential for the development of effective antiviral strategies against these viruses.

## 1. Introduction

Enterovirus A71 (EV71), coxsackievirus A16 (CVA16), and coxsackievirus B3 (CVB3) are members of the Picornaviridae family [1]. EV71 and CVA16 are responsible for hand, foot, and mouth disease (HFMD), with EV71 being particularly responsible for causing severe illness and fatalities [1,2]. CVB3 is responsible for various human diseases, including viral myocarditis [3] and pancreatitis [4,5]. Human EV71, CVA16, and CVB3 infections are complex and involve interactions with host cells and immune responses. Although substantial progress has been made in understanding viral replication and virulence factors, some aspects, such as virus–host interactions, require further investigation. Therefore, understanding the pathogenesis of these viruses and developing effective antiviral strategies is critical.

Recently, specific antiviral compounds targeting EV71, CVA16, and CVB3 have shown significant advancements. Preclinical studies have identified various inhibitors that target different stages of the viral replication cycle. One notable example is pleconaril, a broad-spectrum antiviral agent initially developed for enterovirus (EV) and rhinovirus (RV) infections that has demonstrated efficacy against EV71 and CVB3 [6,7]. Pleconaril inhibits viral attachment and entry into host cells by binding to a hydrophobic pocket of the viral capsid protein, thereby hindering viral replication [8,9]. Clinical trials and studies have demonstrated the potential of pleconaril in reducing the severity and duration of EV and RV infections, although its development was halted owing to adverse effects [10,11,12]. In addition, NITD008, which inhibits RNA polymerase activity; rupintrivir (AG7088), which targets 3Cpro; and GPP3, which blocks virus entry, have shown antiviral activity against diverse EVs and coxsackieviruses [1,12,13]. Despite these findings, none of these inhibitors have progressed to clinical use.

*Saururus chinensis*, a medicinal herbaceous plant widely distributed in Eastern Asian countries and North America, is traditionally used to treat various medical conditions [14,15,16]. Extracts from *S. chinensis* have diverse pharmacological effects, including antiinflammatory, antioxidant, antiasthmatic, antihypertensive, antiangiogenic, and antiapoptotic properties [17,18,19]. Several compounds, including lignans, diterpenes, alkaloids, tannins, flavonoids, steroids, and lipids, have been isolated from *S. chinensis*, and lignan-type compounds, such as sauchinone and manassantin A and B, are considered bioactive [20,21,22,23].

Manassantin B, a compound isolated from *S. chinensis*, activates the stimulator of interferon genes (STING)/TANK-binding kinase-1 (TBK1)/interferon regulatory factor-3 (IRF3) signaling pathway and exhibits antiviral activity against CVB3 [24]. In this study, saucerneol, another compound isolated from *S. chinensis*, displayed significant antiviral activity against EV71, CVA16, and CVB3 infections in Vero cells. The antiviral mechanism of saucerneol involves activation of the STING/TBK-1/IRF3 signaling pathway and an increase in mitochondrial ROS (mROS) generation. Additionally, saucerneol inhibited CVB3 replication in mouse pancreas and mitigated CVB3-induced pancreatitis. These findings emphasize the potential of saucerneol as a natural antiviral agent derived from *S. chinensis*, which is effective against various enteroviral infections, including EV71, CVA16, and CVB3. This research is particularly relevant, as it presents an alternative approach to combating viral pathogens, especially at a time when resistance to existing antiviral drugs is an increasing concern. The natural derivation of saucerneol also suggests the possibility of a better safety profile, which is often a challenge with synthetic compounds. Therefore, we anticipate that our findings will be of great interest to those involved in the search for novel antiviral therapeutics and could lead to the development of more effective treatments for EV infections.

## 2. Materials and Methods

### 2.1. Extraction and Isolation

The dried aerial parts of *S. chinensis* (1.2 kg) were finely ground using a blender. *S. chinensis* powder was macerated in 3 L of methanol at 20 °C for 3 days and filtered (Whatman No. 2). This process was repeated three times using 3 L of methanol. The combined filtrates were evaporated using a 40 °C water bath to yield a dark-green residue (84.2 g) upon removal of the solvent using a rotary evaporator. The total extract was dissolved in 1 L water and then partitioned with *n*-hexane, ethyl acetate (EtOAc), and n-butanol, sequentially. The EtOAc-soluble extract (30.1 g) was subjected to reverse-phase column chromatography (Merck, Darmstadt, Germany, 40–63 μm, 300 g) and eluted with a gradient consisting of methanol:water (2:8, 4:6, 6:4, 8:2, 10:0; 2 × 500 mL each), producing 10 sub-fractions (Fr. 1–10). Among the n-hexane, ethyl acetate (EtOAc), and n-butanol fraction samples, the EtOAc fraction of *S. chinensis* showed the lowest IC_50_ value of 4.59 μg/mL against CVB3 infection in Vero cells. Based on bioassay-guided fractionation, Fr. 7 and 10, which showed the most potent inhibitory effects, were subjected to column chromatography. Fr. 7 was purified by preparative high-performance liquid chromatography (HPLC) using a gradient of 30–100% acetonitrile in water (Shiseido-Capcell Pak C18 UG120, 250 × 10 mm, 10 μm, Tokyo, Japan) to obtain the active compound (Figure S1). 

### 2.2. Plant Material

The antiviral activities of 67 plant extracts were screened against CVB3 using a sulforhodamine B (SRB) assay (data not shown). Among the samples, the methanol extract of *S. chinensis* was confirmed to exhibit approximately 90% antiviral activity against CVB3 at a concentration of 2 μg/mL (Figure S2). Aerial parts of *S. chinensis*, identified by Professor Yong Soo Kwon at Kangwon National University’s College of Pharmacy, were deposited in the university’s herbarium (KNUSC-01) for antiviral compound isolation.

### 2.3. Identification of Active Compound Isolated from S. chinensis

Among the 10 subfractions obtained from the EtOAc fraction of *S. chinensis*, Fr. 7 displayed the strongest antiviral activity. Using SRB bioassay guidance, the active compound was isolated from Fr. 7. The structure of the active compound was confirmed using spectroscopic experiments, including UV, EI-MS, ^1^H-NMR, and ^13^C-NMR. The nuclear resonance signals of ^1^H-NMR and ^13^C-NMR of the active compound were identified using saucerneol (Figure S3).

### 2.4. Apparatus and Reagents

^1^H- and ^13^C-NMR spectral data were recorded in CDCl_3_ on a Bruker DRX 300 spectrometer (Karlsruhe, Germany) at 600 and 150 MHz, respectively, and chemical shifts were presented as δ (ppm). UV spectra were obtained in methanol using a UVICON 933/934 spectrophotometer (Kontron Instrument, Milan, Italy), and mass spectra were obtained via direct infusion or liquid chromatographic introduction into a Finnigan LCQ Advantage MAX ion trap mass spectrometer equipped with a Finnigan Surveyor Modular HPLC system (Thermo Electron Co., Waltham, MA, USA). Silica gel 60 RP-18 (40–63 μm, Merck, Darmstadt, Germany) was used for column chromatography. Pre-coated silica gel plates (silica gel 60 F254, 0.25 and 0.5 mm thickness, Merck) were used for analytical thin-layer chromatography (TLC). Hitachi L-6200 HPLC (Hitachi, Tokyo, Japan) was used to isolate the active compounds (Shiseido Capcell Pak C18 UG120, 250 × 10 mm, 10 μm, Japan).

### 2.5. Cell Culture and Viruses

CVB3, EV71, and CVA16 (obtained from the American Type Culture Collection, Manassas, VA, USA) were propagated at 37 °C in Vero cells (ATCC), a kidney epithelial cell line originally derived from an African green monkey. Vero cells were cultured in Dulbecco’s modified eagle’s medium (DMEM) supplemented with 10% heat-inactivated fetal bovine serum and 0.01% antibiotic–antimycotic solution in a 37 °C incubator with 5% CO_2_ (SANYO Electric Co, Osaka, Japan). Antibiotic–antimycotic solution, trypsin–EDTA, fetal bovine serum, and DMEM were provided by Corning (Corning Incorporated, Corning, NY, USA). Tissue culture plates were purchased from Falcon (BD Biosciences, Franklin Lakes, NJ, USA).

### 2.6. Antiviral Activity Assay

Antiviral activity and cytotoxicity were evaluated using the SRB assay using cytopathic effects (CPE). One day prior to infection, 3 × 10^4^ cells/well were seeded in a 96-well culture plate. The following day, the culture medium was aspirated and the cells were washed with 1× phosphate-buffered saline (PBS) (Corning Incorporated, Corning, NY, USA). The infectivity of each virus was determined using the SRB method to monitor CPE, allowing the percentage of cell viability to be determined. Based on the determined mammalian cell viability for each virus, 0.09 mL of the diluted virus suspensions of CVB3, EV71, and CVA16 were added to mammalian cells. This dose was selected to produce appropriate CPEs 48 h after infection. The antiviral activity of each test compound was determined using five-fold diluted concentrations ranging from 0.4 to 50 μg/mL. We used three wells each for the viral control (virus-infected and non-drug-treated cells) and cell controls (non-infected and non-drug-treated cells). Rupintrivir and SRB were purchased from Sigma-Aldrich (Burlington, MA, USA). The 96-well culture plates were incubated at 37 °C in 5% CO_2_ for 2–3 days until 70–80% CPE was observed. The supernatant was discarded, and the wells were thoroughly washed twice with PBS at the end of the culture. Cells were fixed with ice-cold 70% acetone (100 μL/well) and stained with 0.4% SRB in 1% acetic acid. The absorbance was measured at 562 nm using a SpectraMax^®^ i3 microplate reader (Molecular Devices, Palo Alto, CA, USA) with a reference absorbance of 620 nm. The results were then transformed into percentages of the controls, and the percent protection achieved by the test compound in virus-infected cells was calculated using the following formula: ((OD_t_)_virus_ − (OD_c_)_virus_) ÷ ((OD_c_)_mock_ − (OD_c_)_virus_) × 100%, where (OD_t_)_virus_ is the optical density measured with a given concentration of the test compound in virus-infected cells; (OD_c_)_virus_ is the optical density of the non-drug-treated, virus-infected control cells; and (OD_c_)_mock_ is the optical density.

### 2.7. Quantitative RT-PCR (RT-qPCR)

Total RNA was extracted from Vero cells and pancreata of mice using a QIAamp^®^ viral RNA mini kit (Qiagen, Hilden, Germany). Taqman real-time PCR and reverse transcription PCR were carried out using AgPath-ID™ One-Step RT-PCR Reagents (Applied Biosystems, Waltham, MA, USA) and a Bio-Rad CFX96 thermal cycler (Bio-Rad, Hercules, CA, USA). The EV71 5′ noncoding region (NCR) of the gene was detected using qRT-PCR. The following EV71 5′NCR primers were used: forward primer 5′-GCGATTGTCACCATWAGCAGYCA-3,’ reverse primer 5′-GGCCCCTGAATGCGGCTAATCC-3,’ and probe primer 5′-CCGACTACTTTGGGWGTCCGTGT-3′. The following *GAPDH* primers were used: forward primer 5′-GGTCTCCTCTGACTTCAACA-3′, reverse primer 5′-AGCCAAATTCGTTGTCATAC-3′, and probe primer 5′-CCCTCAACGACCACTTTGTCAAG-3′. The cycling conditions were as follows: heating at 45 °C for 10 min for reverse transcription, reverse transcription inactivation, and initial denaturation at 95 °C for 10 min, followed by 40 cycles of amplification at 95 °C for 15 s and at 62 °C for 45 s. The results were analyzed using the real-time system AB 7900HT software (Life Technologies) and all values were normalized to GAPDH levels. A Bio-Rad CFX96 thermal cycler was used at the Core Facility for Innovative Cancer Drug Discovery (CFICDD) at Kangwon National University.

### 2.8. Immunofluorescence

Vero cells (5 × 10^5^ cells/well) were seeded in six-well culture plates. After 24 h, the cells were infected with EV71 and treated with 10 μg/mL saucerneol for 24 h in DMEM containing 1% FBS. The cells were washed with PBS and fixed with 4% paraformaldehyde (T&I chem, Incheon, South Korea) for 15 min, permeabilized with 0.25% Triton-X 100 (Sigma Aldrich), and blocked with 1% BSA (MP Biomedicals, Solon, OH, USA) for 1 h at 20 °C. Cells were stained using anti-VP3 antibodies (Invitrogen, Carlsbad, CA, USA) as the primary antibody, incubated overnight at 4 °C, washed with PBS, stained with goat anti-mouse IgG H&L (Alexa Fluor^®^ 647) (Abcam, Cambridge, UK) secondary antibodies, and incubated for 2 h at 20 °C. After washing with PBS, the cells were stained with 4′,6-diamidino-2-phenylindole (DAPI) and analyzed using an LSM 880 with Airyscan (Carl Zeiss, Oberkochen, Land Baden-Württemberg, Germany).

### 2.9. Time-of-Addition Assay

A total of 10 μg/mL saucerneol and 2 μg/m rupintrivir were added to Vero cells either before (−1 h), during (0 h), or after (1, 2, 4, 6, 8, and 10 h) infection with EV71. Total RNA was isolated at the indicated time points post infection, and 5′NCR gene levels in EV71 and CVA16 were analyzed using real-time PCR.

### 2.10. Time-Course Assay

Vero cells infected with EV71 and CVA16 were harvested at the indicated time points, including 4, 6, 8, 10, and 12 h post infection. Saucerneol (10 μg/mL) and rupintrivir (2 μg/mL) were added at the time of infection. Total RNA was isolated at the indicated time points post infection, and 5′NCR gene levels in EV71 and CVA16 were analyzed using real-time PCR.

### 2.11. mROS Measurement

Vero cells (1 × 10^6^ cells/well) were seeded in six-well plates. After 24 h, the supernatant of each well was replaced with viral infection medium containing saucerneol. After 4 h, the cells were harvested using trypsin-EDTA and stained with Mito-Sox Red (Invitrogen), a mitochondrial superoxide indicator, according to the manufacturer’s guidelines. Stained cells were analyzed using FACSVerse (BD Biosciences, Franklin Lakes, NJ, USA) without formalin fixation. FACSVerse was performed at the CFICDD at Kangwon National University.

### 2.12. Western Blot

Total protein lysates from cells were prepared using sonication with PRO-PREP™ Protein Extraction Solution (iNtRON Biotechnology, Seongnam, South Korea). Protein levels were determined using a Pierce™ BCA Protein Assay Kit (Thermo Fisher Scientific, Waltham, MA, USA). Equivalent amounts of proteins were separated by sodium dodecyl sulfate-polyacrylamide gel electrophoresis and transferred to membranes. The membranes were subsequently incubated with primary antibodies for 24 h at 4 °C. Primary antibodies: anti-VP3, β-actin (Santa Cruz Biotechnology, Dallas, TX, USA), STING, Phospho-STING, TBK1/NAK, Phospho-TBK1/NAK, IRF3, Phospho-IRF3 (Cell Signaling, Denver, MA, USA). Thereafter, secondary Abs, goat anti-mouse IgG F(ab′)2, polyclonal Abs (HRP conjugated) (Enzo Life Sciences, Farmingdale, NY, USA), and anti-rabbit IgG (HRP-linked antibody) (Cell Signaling, Denver, MA, USA), were added for 2 h at 20 °C. Proteins were detected using West Femto Maximum Sensitivity Substrate (Abbkine, Atlanta, GA, USA) and visualized using an ImageQuant LAS 4000 Mini System (Cytiva, Marlborough, MA, USA). Chemiluminescence intensity was analyzed using ImageJ software (NIH, Bethesda, MD, USA).

### 2.13. Animal Model

Five-week-old female BALB/c mice were purchased from the SPL laboratory animal company (Koatech Bio, Pyeongtaek, South Korea). All mice were maintained under specific pathogen-free conditions for 1 week in the experimental facilities at Kangwon National University (Chuncheon, South Korea), where they received sterilized food and water ad libitum and were housed at 20–22 °C with a 12 h light/dark cycle. All animal experiments were performed in accordance with the guidelines set and approved by the Institutional Animal Care and Use Committee of Kangwon National University (KW-161101-2). Six-week-old female BALB/c mice were intraperitoneally injected with 1 × 10^6^ PFU/mouse CVB3. BALB/c mice infected with CVB3 received intraperitoneal administration of saucerneol (2 mg/kg/day) for 9 days.

### 2.14. Histological Analysis

The tissues from CVB3-infected mice were fixed with 4% formaldehyde (DANA Korea, Incheon, South Korea) overnight at 20 °C. CVB3-infected mouse tissues were dehydrated in ethanol and xylene and embedded in paraffin. Tissue sections were sliced into 5 μm-thick samples and stained with hematoxylin and eosin (H&E). The stained tissues were evaluated to determine the degree of inflammation using an Ocus^®^40 microscope scanner (Grundium, Tampere, Finland).

### 2.15. Statistical Analysis

To compare multiple groups, we performed one-way analysis of variance (ANOVA) followed by Bonferroni’s multiple comparison test. Statistical analyses were performed using GraphPad Prism software version 5 (GraphPad Software, Boston, MA, USA). Results with *p*-values of less than 0.05 were considered statistically significant.

## 3. Results

### 3.1. Antiviral Activity of Saucerneol Isolated from S. chinensis against EV

**Figure 1:**
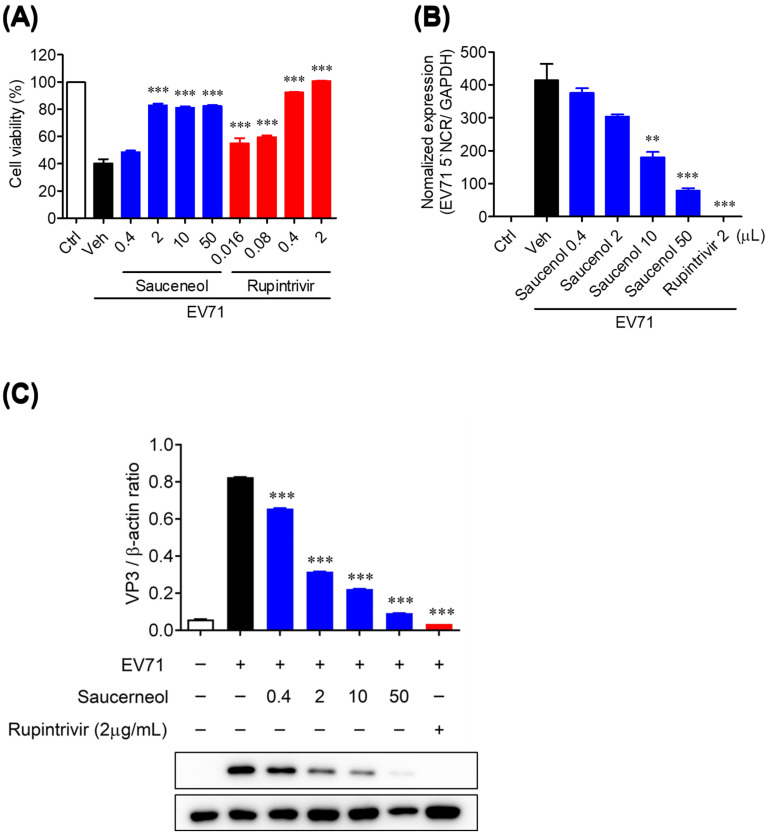
Antiviral activity of saucerneol against enterovirus A71 (EV71) in vitro. (**A**) Vero cells were infected with 5 × 10^3^ PFU EV71 and treated with saucerneol. Cell viability and cytotoxicity by saucerneol treatment were measured using the sulforhodamine B (SRB) assay. (**B**) Relative EV71 5′ noncoding region (NCR) gene expression in EV71-infected Vero cells was determined using real-time PCR. (**C**) Western blot analysis of VP3 in EV71-infected cells after treatment with the vehicle or 0.4–50 µg/mL of saucerneol for 24 h. The ratio of VP3 to β-actin based on the quantification of bands in the immunoblot. Rupintrivir was used as a positive control. For the validation assessment, (**A**) were obtained through three independent experiments and (**B**,**C**) were obtained through two independent experiments. The results are shown as the mean ± SEM. ** *p* < 0.01 and *** *p* < 0.001 for comparison with the EV71-infected vehicle-treated group (Veh) based on one-way ANOVA with Bonferroni’s multiple comparison test.

To evaluate the antiviral activity of saucerneol, an active compound isolated from *S. chinensis* against EV71, CVA16, and CVB3 cells, a cell viability assay was conducted using virus-infected Vero cells. Saucerneol demonstrated approximately 90% inhibition of CVB3-induced cytotoxicity at a concentration of 2 µg/mL, which was comparable to the positive control, rupintrivir (Figure S4). Saucerneol also showed antiviral activity against EV71 and CVA16 in Vero cells (Figure 1A and Figure S5). 

**Figure 2:**
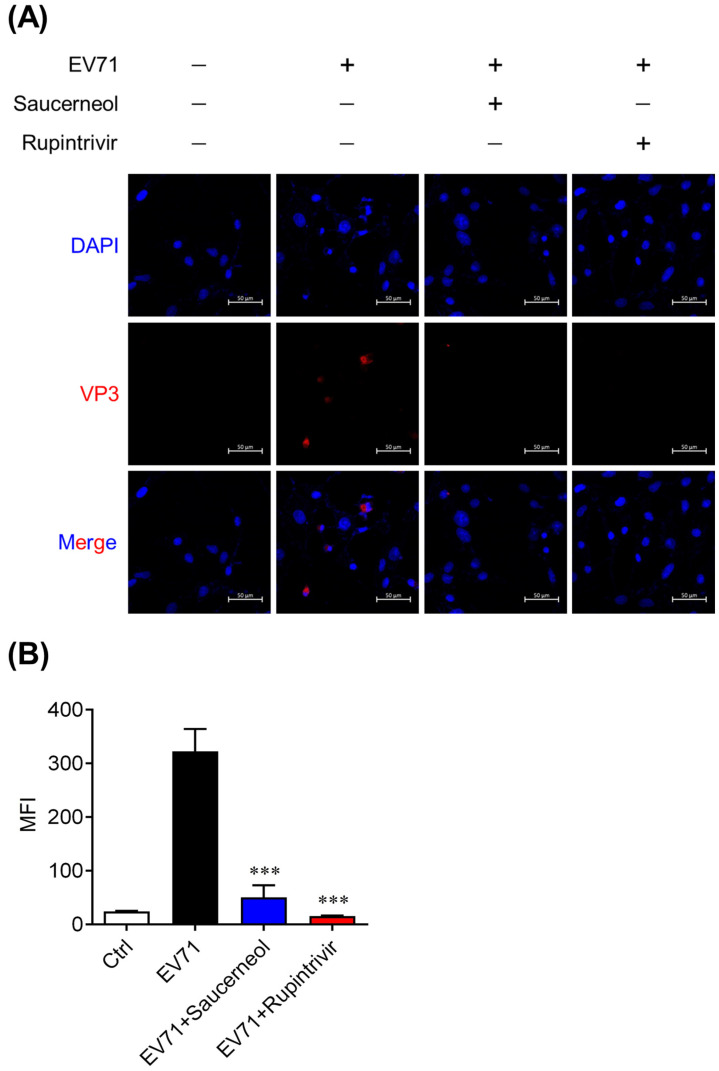
Antiviral effect of saucerneol confirmed using immunofluorescence. (**A**) Representative image of VP3 staining in EV71-infected and non-infected Vero cells treated with 10 µg/mL saucerneol for 24 h. The image was observed under a confocal microscope. Red signals represent VP3; blue signals represent DAPI-stained nuclei. Scale bar, 50 µm. (**B**) MFI values of immunofluorescence images were quantified using ZEN software. The data were obtained through two independent experiments for validation assessment. The results are shown as mean ± SEM. *** *p* < 0.001 for comparison with the EV71-infected vehicle-treated group (Veh) based on one-way ANOVA with Bonferroni’s multiple comparison test.

To confirm whether saucerneol inhibits the replication of EV71 in Vero cells, real-time PCR was used, and we found that saucerneol inhibited the viral genome by more than 50% at a concentration of 10 µg/mL (Figure 1B). Additionally, saucerneol strongly suppressed the expression of VP3 protein of EV71 at a concentration of 2 µg/mL (Figure 1C), suggesting that saucerneol strongly inhibits the viral replication and protein translation stages, which are the initial stages of the EV71 life cycle. Next, we confirmed the antiviral activity of saucerneol using confocal microscopy. As a result of analyzing the antiviral activity using immunofluorescence staining of the VP3 protein, the capsid protein of EV71, 24 h after viral infection, saucerneol significantly inhibited the viral VP3 protein to the same level as rupintrivir, the positive control (Figure 2). 

Overall, these results demonstrate the potential of saucerneol as an effective antiviral agent against EV71, CVA16, and CVB3.

### 3.2. Time-of-Addition and Time-Course Assay of Saucerneol in Virus-Infected Cells

**Figure 3:**
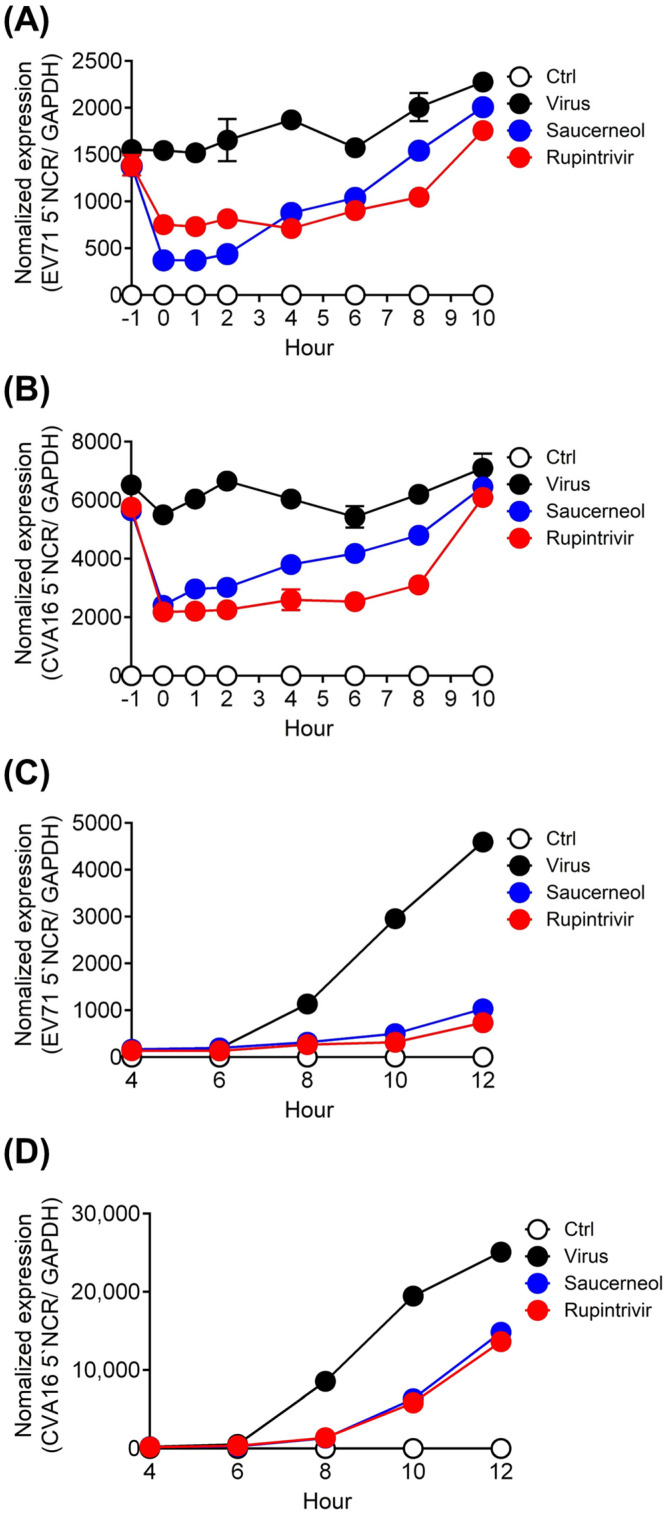
Saucerneol inhibited EV71 and coxsackievirus A16 (CVA16) replication at the early stage of viral infection. (**A**) Vero cells were treated with 10 µg/mL saucerneol during or after infection with EV71 at the indicated time points and viral mRNA was analyzed 14 h post infection. (**B**) Vero cells were treated with 10 µg/mL saucerneol during or after infection with CVA16 at the indicated time points and viral mRNA was analyzed 14 h post infection. (**C**) Vero cells were infected with EV71 and harvested at the indicated time points, including at 4, 6, 8, 10, and 12 h after treatment with 10 µg/mL saucerneol. (**D**) Vero cells were infected with 1 × 10^3^ PFU CVA16 and harvested at the indicated time points, including at 4, 6, 8, 10, and 12 h after treatment with 10 µg/mL saucerneol. Rupintrivir was used as a positive control. The data were obtained through two independent experiments for validation assessment. Total RNA was isolated and viral RNA was analyzed using reverse transcription–real-time PCR.

To identify the mechanism underlying the antiviral activity of saucerneol, we conducted time-of-addition and time-course assays for EV71 and CVA16. The time-of-addition assay showed that the antiviral effect of saucerneol decreased depending on the time interval between infection and treatment. A potent antiviral effect was observed during the early stages of viral infection (0–2 h), with a marginal antiviral effect extending for up to 4–6 h (Figure 3A,B). However, when the drug was administered before the viral infection, no antiviral effects were observed. Additionally, using a time-course assay, we confirmed that saucerneol continuously inhibited EV71 and CVA16 infections. Similar to rupintrivir, a 3C protease inhibitor, saucerneol, strongly inhibited the expression of viral RNA beginning at 8 h, when viral replication increased (Figure 3C,D). Our results suggest that saucerneol exhibits antiviral activity against EV71 and CVA16 primarily during the early stages of infection and shares similarities with rupintrivir in potentially inhibiting viral replication and translation.

### 3.3. Saucerneol Induced Mitochondrial Damage with mROS Generation

**Figure 4:**
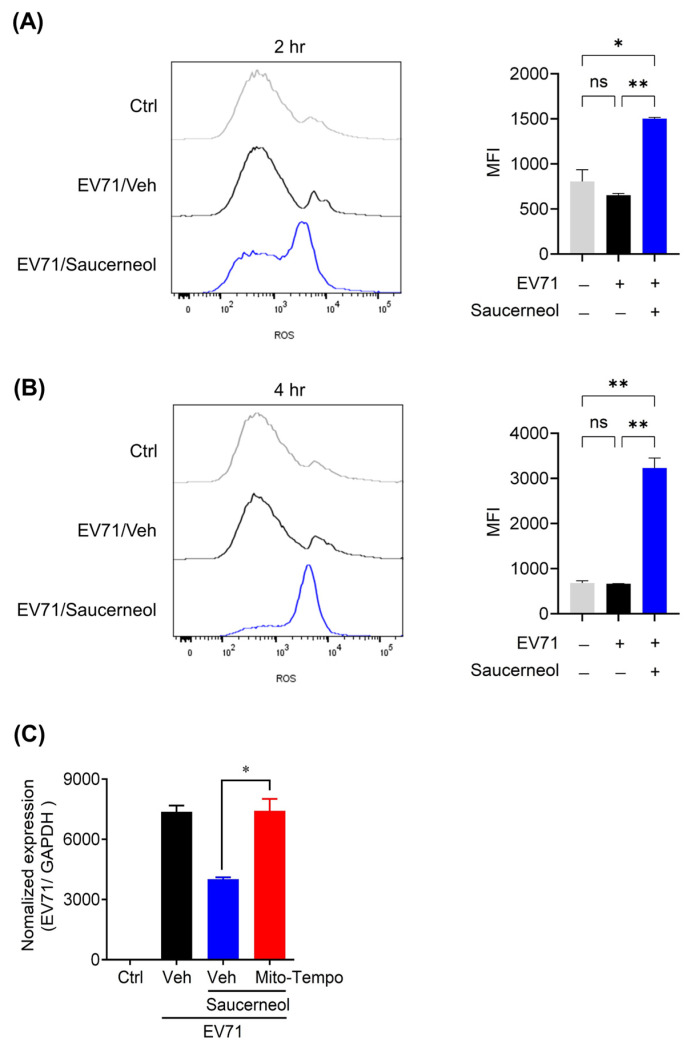
Saucerneol induced mitochondrial ROS. (**A**) EV71-infected Vero cells were treated with 10 µg/mL saucerneol. After 2 h of infection and treatment, harvested cells were stained with 5 µM of Mito-SOX reagent and analyzed using fluorescence-activated cell sorting (FACS) to detect reduction in mitochondrial ROS. The mean Mito-SOX fluorescence index (MFI) was measured. (**B**) EV71-infected Vero cells were treated with 10 µg/mL saucerneol. After 4 h of infection and treatment, harvested cells were stained with 5 µM of Mito-SOX reagent and analyzed by fluorescence-activated cell sorting (FACS) to detect any reduction in mitochondrial ROS. The Mito-SOX MFI was measured. (**C**) Mito-TEMPO (50 µM) was added to saucerneol-treated and EV71-infected cells. For the validation assessment, (**A**,**B**) were obtained through two independent experiments and (**C**) were obtained through three independent experiments. The results are shown as mean ± SEM. * *p* < 0.05, ** *p* < 0.01 and ns (not significant) for comparison with each group based on one-way ANOVA with Bonferroni’s multiple comparison test.

In a previous study, we reported that manassantin B increased mROS levels in Vero cells infected with CVB3 [24]. Thus, in this study, we investigated whether saucerneol, which possesses a moiety structurally similar to that of manassantin B, has a similar effect on mROS generation in Vero cells infected with EV71. We treated EV71-infected Vero cells with saucerneol and performed flow cytometry to measure mROS levels at 2 and 4 h post treatment. Treatment of EV71-infected Vero cells with saucerneol led to a significant increase in mitochondrial mROS levels. Specifically, mROS levels more than doubled after 2 h of treatment (Figure 4A) and increased by approximately five-fold after 4 h of treatment (Figure 4B). To further explore whether the antiviral activity of saucerneol was indeed mediated by mROS, we treated Vero cells infected with EV71 with a mitochondria-targeted antioxidant, Mito-TEMPO (Enzo Biochem, Farmingdale, NY, USA), at a concentration of 50 μM. This treatment significantly inhibited the antiviral activity of saucerneol (Figure 4C). In addition, Mito-TEMPO inhibited the antiviral activity of saucerneol in Vero cells infected with CVA16 (Figure S6). These results suggest that the antiviral activity of saucerneol against EV71 and CVA16 is closely linked to increased mROS levels.

### 3.4. Saucerneol Triggered the STING/TBK-1/IRF3 Signaling Pathway in Virus-Infected Cells

**Figure 5:**
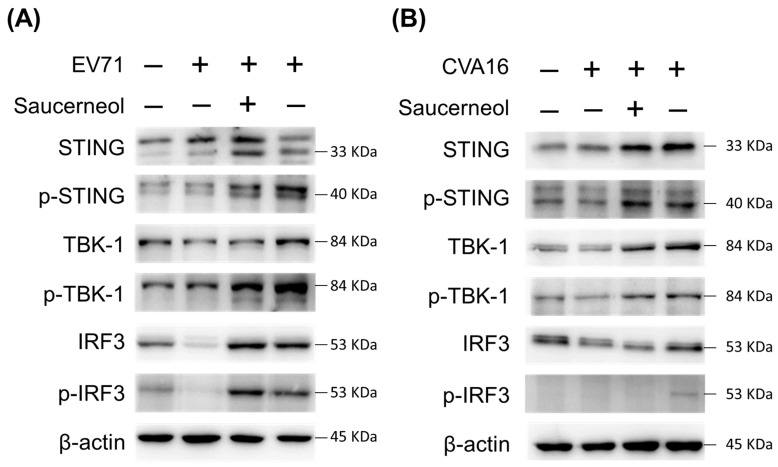
Saucerneol induced the STING/TBK-1/IRF3 signaling pathway in Enterovirus 71- and Coxsackievirus A16-infected cells. (**A**) Representative immunoblot results of STING, p-STING, TBK-1, p-TBK-1, IRF3, p-IRF3, and β-actin to evaluate activation of their expression in (A) EV71 and (**B**) CVA16 -infected Vero cells or uninfected cells after treatment with saucerneol (10 µg/mL). The samples were derived from the same experiment, and gels/blots were processed in parallel.

Cytoplasmic mtDNA activates cyclic GMP–AMP synthase (cGAS), initiating the STING/TBK-1/IRF3 pathway essential for antiviral gene expression [25,26]. Western blot analysis revealed a significant increase in the expression levels of STING, p-STING, TBK-1, p-TBK-1, IRF3, and p-IRF3 in Vero cells infected with EV71 (Figure 5A) and CVA16 (Figure 5B) following saucerneol treatment. These levels notably surpassed those in the virus-infected group, normalized against β-actin levels. Furthermore, a conspicuous rise in STING and TBK-1 phosphorylation was evident in cells treated solely with saucerneol compared to untreated control cells (Figures S7 and S8). However, treatment of virus-infected cells with saucerneol did not substantially elevate the ratios of p-STING to STING, p-TBK-1 to TBK-1, or p-IRF3 to IRF3, potentially owing to an overall increase in relevant protein levels. Hence, these findings suggest saucerneol’s role in activating the STING/TBK-1/IRF3 pathway by enhancing the translation of STING, TBK-1, and IRF3 and by inducing STING and TBK-1 phosphorylation in infected cells following saucerneol treatment, highlighting its potential in effective antiviral defense against EV71 (Figure 5A) and CVA16 (Figure 5B, Figures S7 and S8).

### 3.5. Saucerneol Inhibits CVB3 Replication and Pancreas Inflammation In Vivo

**Figure 6:**
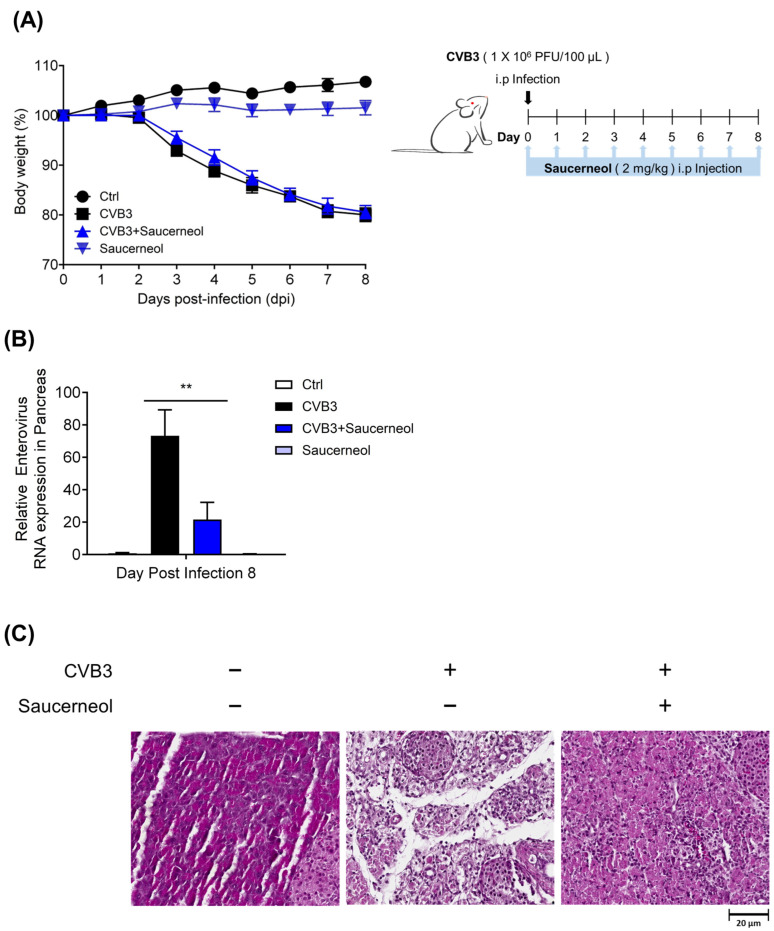
Antiviral activity of saucerneol against coxsackievirus B3 (CVB3) in vivo. (**A**) BALB/c mice were infected intraperitoneally with a 1 × 10^6^ PFU of CVB3 and then assessed by body weight. Saucerneol treatment was administered intraperitoneally at 2 mg/kg daily until the mice were euthanized 8 days post infection. (**B**) Enterovirus RNA levels in mice pancreas treated with saucerneol. RNA was isolated and viral RNA was analyzed using reverse transcription–real-time PCR. (**C**) Representative hematoxylin and eosin (H&E)-stained images of pancreas sections showing uninfected, CVB3-infected, and CVB3-infected groups treated with saucerneol (scale bar = 20 μm). The data were obtained through two independent experiments for validation assessment. ** *p* < 0.01 for comparison with the CVB3-infected group based on one-way ANOVA with Bonferroni’s multiple comparison test.

To assess the antiviral effects of saucerneol in vivo, BALB/c mice were intraperitoneally injected with CVB3. Initially, we monitored body weight changes in CVB3-infected mice treated with saucerneol for 8 days following infection. CVB3-infected mice experienced an approximately 20% decrease in body weight over the eight-day period, and the administration of saucerneol did not significantly prevent weight loss attributed to CVB3 infection (Figure 6A). Next, considering that the pancreas is a well-known target organ of CVB3, we evaluated CVB3 gene expression and CVB3-induced pancreatitis in mice [27]. Eight days after CVB3 infection, we analyzed the viral RNA in the mouse pancreas using RT-PCR. The results indicate that there was a significant reduction in viral RNA in the group treated with saucerneol compared to that in the CVB3-infected group (Figure 6B). Furthermore, histological analysis of the mouse pancreas revealed that, in uninfected mice, the pancreatic tissue appeared to be histologically normal. However, in mice infected with CVB3 for 8 days, almost all acinar cells were depleted and inflammatory cells infiltrated the tissue. In CVB3-infected mice, a major portion of the pancreas was damaged by CVB3 infection; however, partially undamaged acinar cells could still be identified (Figure 6C).

## 4. Discussion

One of the lignans isolated from *S. chinensis*, saucerneol, exhibits various pharmacological activities. In this study, we identified saucerneol as an effective antiviral compound against EV71, CVA16, and CVB3 in vitro and confirmed its antiviral efficacy against CVB3 in vivo. Currently, no antiviral agents are clinically approved for the treatment of hand, foot, and mouth diseases caused by enteric viruses. The development of new antiviral drugs against these viruses is an urgent challenge. 

Pleconaril is a broad-spectrum antiviral compound that inhibits enterovirus and human rhinovirus capsid proteins [28]. However, owing to inconsistent results in the treatment of enteroviral infections and limited clinical efficacy against the common cold caused by HRV, clinical development of pleconaril was halted in 2002 [29]. Rupintrivir, a 3C protease inhibitor of enteroviruses, exhibits broad-spectrum antiviral activity against members of the Picornaviridae family. Nevertheless, a V104I-resistant mutation was identified within the 3C protease of enterovirus D68 [30,31,32,33]. Enviroxime, which targets the host protein phosphatidylinositol-4-kinase III β (PI4KIIIβ), is the only enterovirus replication inhibitor that has undergone clinical evaluation [34]. However, it was discontinued in clinical phase II owing to the toxicity observed in clinical trials evaluating its use for the treatment of the common cold associated with HRV [35]. Ribavirin, in the form of ribavirin triphosphate, can serve as a base analog for either ATP or GTP. The antiviral potency of ribavirin has been demonstrated against various RNA viruses, both in vitro and in vivo, including EV71 [36]. However, a recent study showed that ribavirin failed to protect 1-day-old mice infected with EV71 [6]. Furthermore, ribavirin did not significantly prevent EV71 infection in Vero cells and even exhibited high levels of cytotoxicity [37]. This highlights the potential significance of developing new antiviral agents for the treatment of EV71 infections.

Our research has revealed that saucerneol exhibits potent antiviral activity, inhibiting more than 80% of EV71, CVA16, and CVB3 at a concentration of 2 µg/mL. The cytotoxicity of saucerneol on Vero cells was not observed up to 10 µg/mL, indicating the absence of cytotoxicity at saucerneol concentrations below 10 µg/mL. No significant difference in body weight was observed compared to mice that did not receive saucerneol. Notably, no significant toxicity was observed at the administered dose of 2 mg/kg of saucerneol, even in the in vivo setting, during the 9 days of treatment.

Double-stranded DNA generated through viral infection or cellular stress can induce the assembly of cGAS oligomers, leading to the enzymatic activation of cGAS and the production of 2′3′ cyclic GMP–AMP (cGAMP). Subsequently, cGAMP binds to the stimulator of interferon genes (STING) located on the endoplasmic reticulum (ER) membrane, initiating a downstream cascade. cGAMP-bound STING recruits TANK-binding kinase 1 (TBK1), which facilitates the phosphorylation of TBK1 and STING at Ser366. This leads to the recruitment of interferon regulatory factor 3 (IRF3), which, upon phosphorylation by TBK1, undergoes dimerization and translocates to the nucleus. These events trigger the expression of type I interferons, interferon-stimulated genes (ISGs), inflammatory mediators, pro-apoptotic genes, and chemokine genes [38]. Proper activation of this IFNAR signaling cascade is critical for controlling viral replication in the context of EV71, CVB3, CVA16, and related viral infections [39]. However, it is evident that certain viruses have evolved mechanisms to suppress the activation of the IFNAR signaling pathway. Therefore, various strategies can be devised to stimulate these pathways and enhance the effectiveness of antiviral agents.

mROS can disrupt mitochondrial transcription factor A (TFAM) and mtDNA stability, leading to mtDNA release and mitochondrial dysfunction [40]. In our previous study, we reported that manassantin B induces the cytoplasmic release of mtDNA in CVB3-infected cells, activates the cGAS/STING/TBK-1/IRF3 pathway, and enhances IFN-I signaling, thereby exhibiting antiviral activity [24]. In this study, saucerneol significantly increased mROS levels in EV71-infected Vero cells, leading to notable phosphorylation of STING, TBK-1, and IRF3; activation of gene expression; and inhibition of EV71. Additionally, saucerneol suppresses viral replication in CVB3-infected mice and reduces virus-induced pancreatitis.

Therefore, our results suggest that the antiviral activity induced by saucerneol treatment is associated with the activation of the type I IFN signaling pathway through the generation of mROS and activation of the cGAS/STING/TBK-1/IRF3 pathway. Further research, including clinical trials, is needed to fully assess the efficacy and safety of saucerneol for clinical use alongside the established anti-viral compounds. In future, we plan to evaluate the applicability of this strategy for antiviral drug development and continue to search for potential antiviral candidates that induce mROS production through compound screening.

## Data Availability

The data presented in this study are available upon request from the corresponding author.

## Supplementary Materials

The following supporting information can be downloaded at: https://www.mdpi.com/article/10.3390/v16010016/s1, Figure S1: Isolation and purification of active compound; Figure S2: Antiviral activity of *S. chinensis* against CVB3 in vitro; Figure S3: Structure of saucerneol; Figure S4: Antiviral activity of saucerneol against CVB3 in vitro; Figure S5: Antiviral activity of saucerneol against CVA16 in vitro; Figure S6: Antiviral activity of mROS induced by suacerneol against CVA16; Figure S7: Saucerneol induced the STING/TBK-1/IRF3 signaling pathway in Enterovirus 71-infected cells; Figure S8: Saucerneol induced the STING/TBK-1/IRF3 signaling pathway in Coxsackievirus A16-infected cells.
