# Supplementary material for: Antiviral Mechanisms of Saucerneol from Saururus chinensis against Enterovirus A71, Coxsackievirus A16, and Coxsackievirus B3: Role of Mitochondrial ROS and the STING/TKB-1/IRF3 Pathway
Source: Viruses. 2023 Dec 21;16(1):16. doi: 10.3390/v16010016 (PMC10821076; doi:10.3390/v16010016)
Supplement: Supplementary file 1 [file viruses-16-00016-s001.zip › viruses-2738872-supplementary.pptx]

## Slide 1
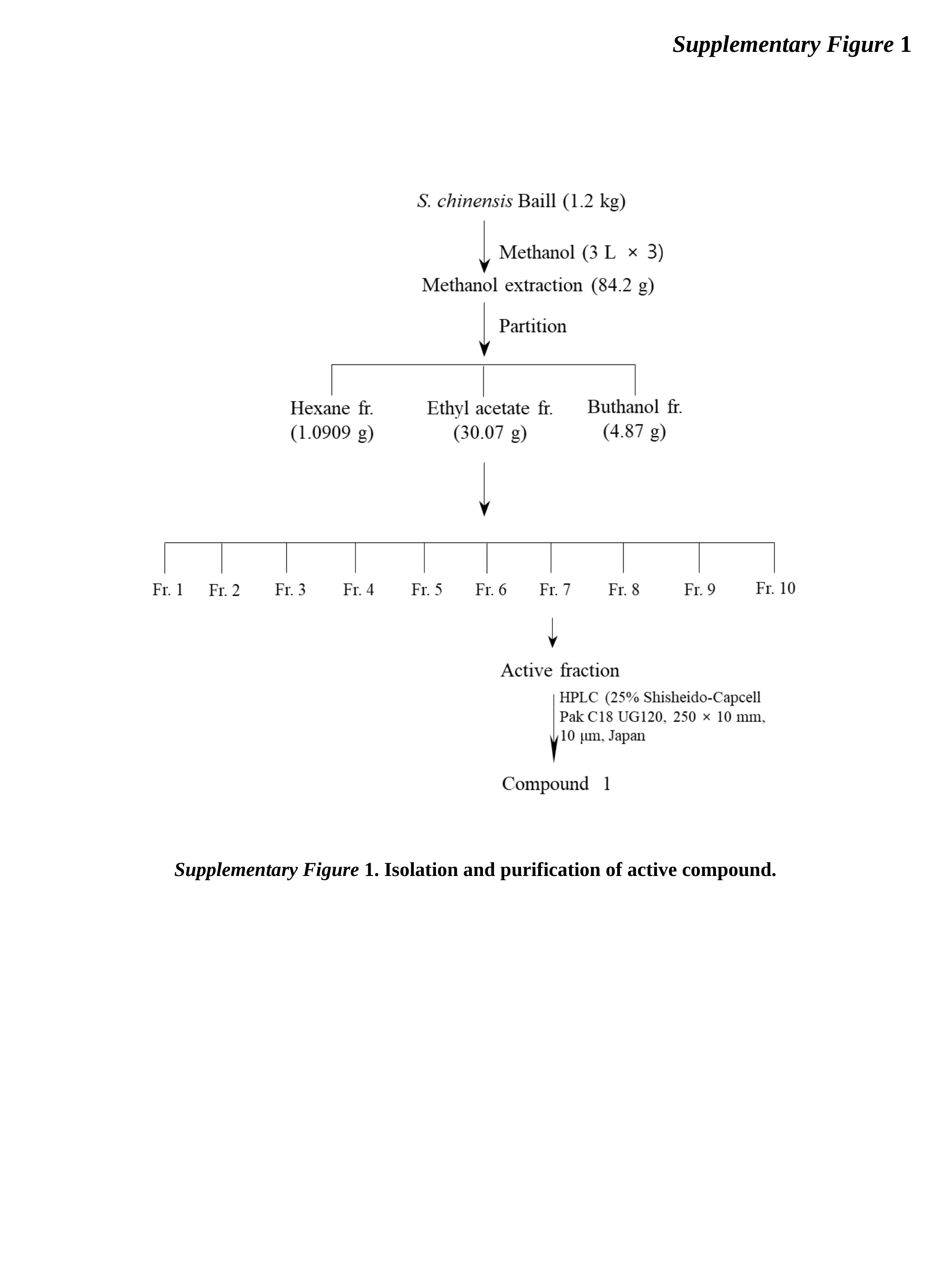

Supplementary Figure 1
Supplementary Figure 1. Isolation and purification of active compound.

## Slide 2
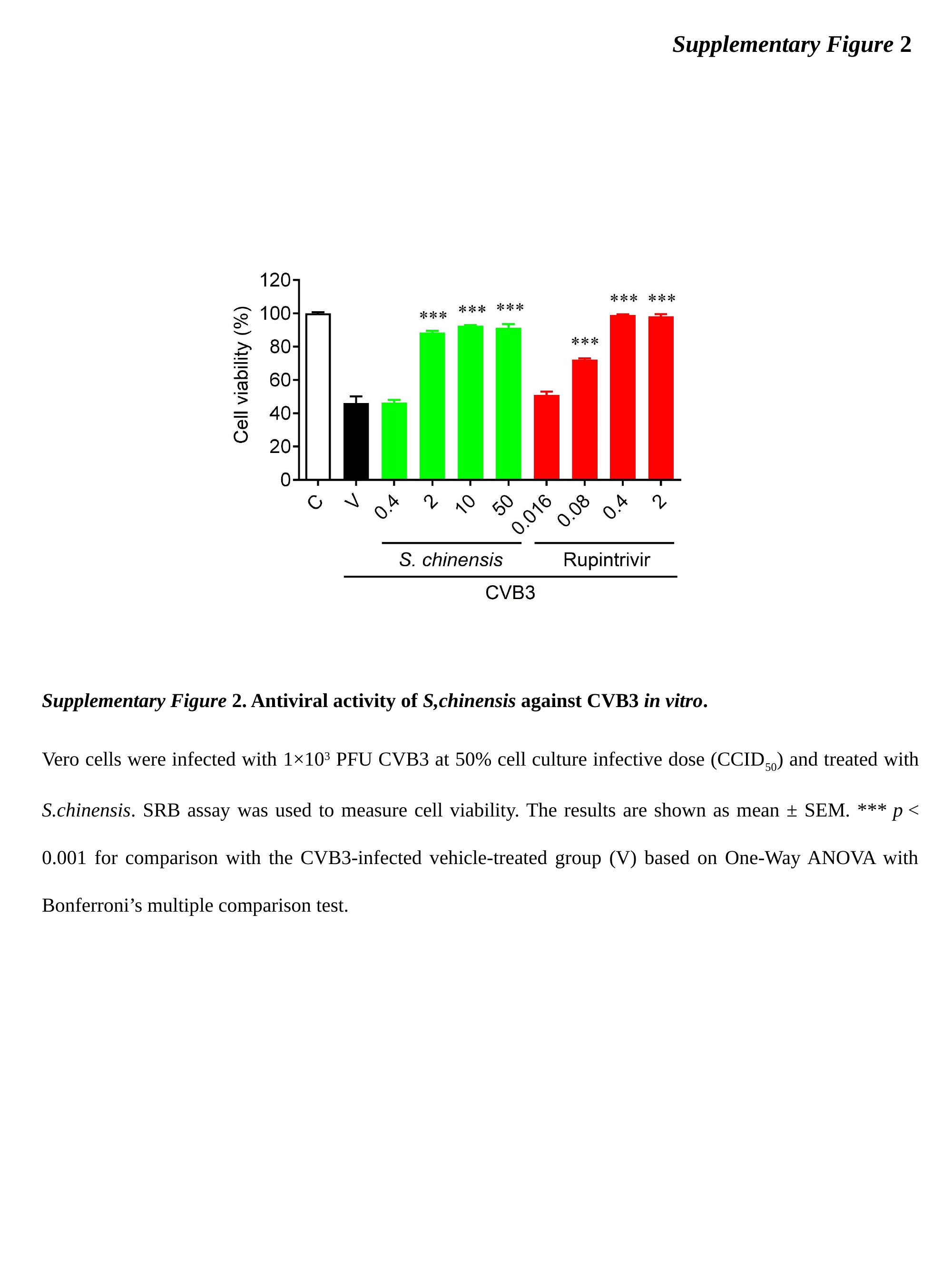

Supplementary Figure 2
Supplementary Figure 2. Antiviral activity of S,chinensis against CVB3 in vitro.
Vero cells were infected with 1×10^3^ PFU CVB3 at 50% cell culture infective dose (CCID50) and treated with S.chinensis. SRB assay was used to measure cell viability. The results are shown as mean ± SEM. *** p < 0.001 for comparison with the CVB3-infected vehicle-treated group (V) based on One-Way ANOVA with Bonferroni’s multiple comparison test.

## Slide 3
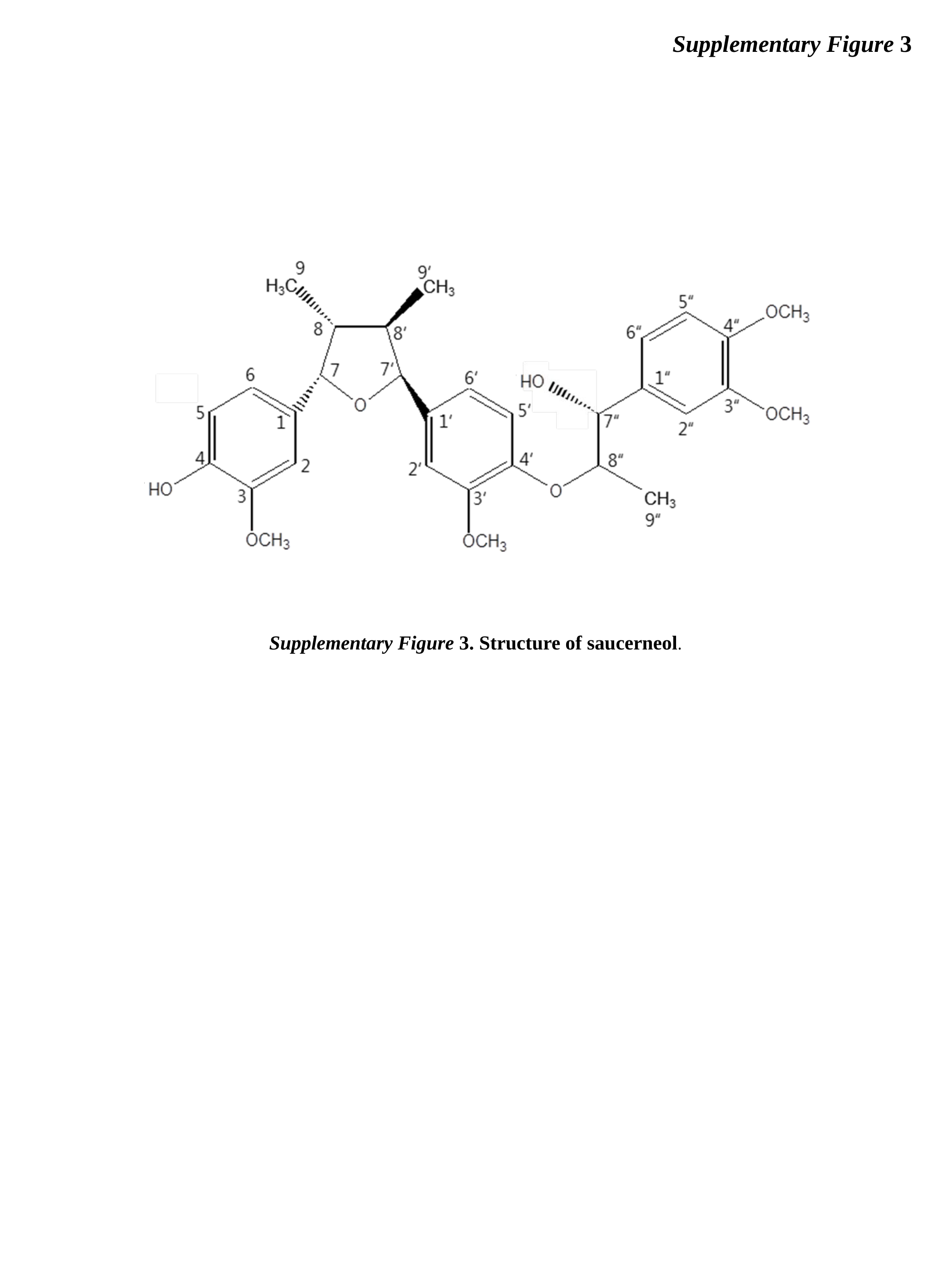

Supplementary Figure 3
Supplementary Figure 3. Structure of saucerneol.

## Slide 4
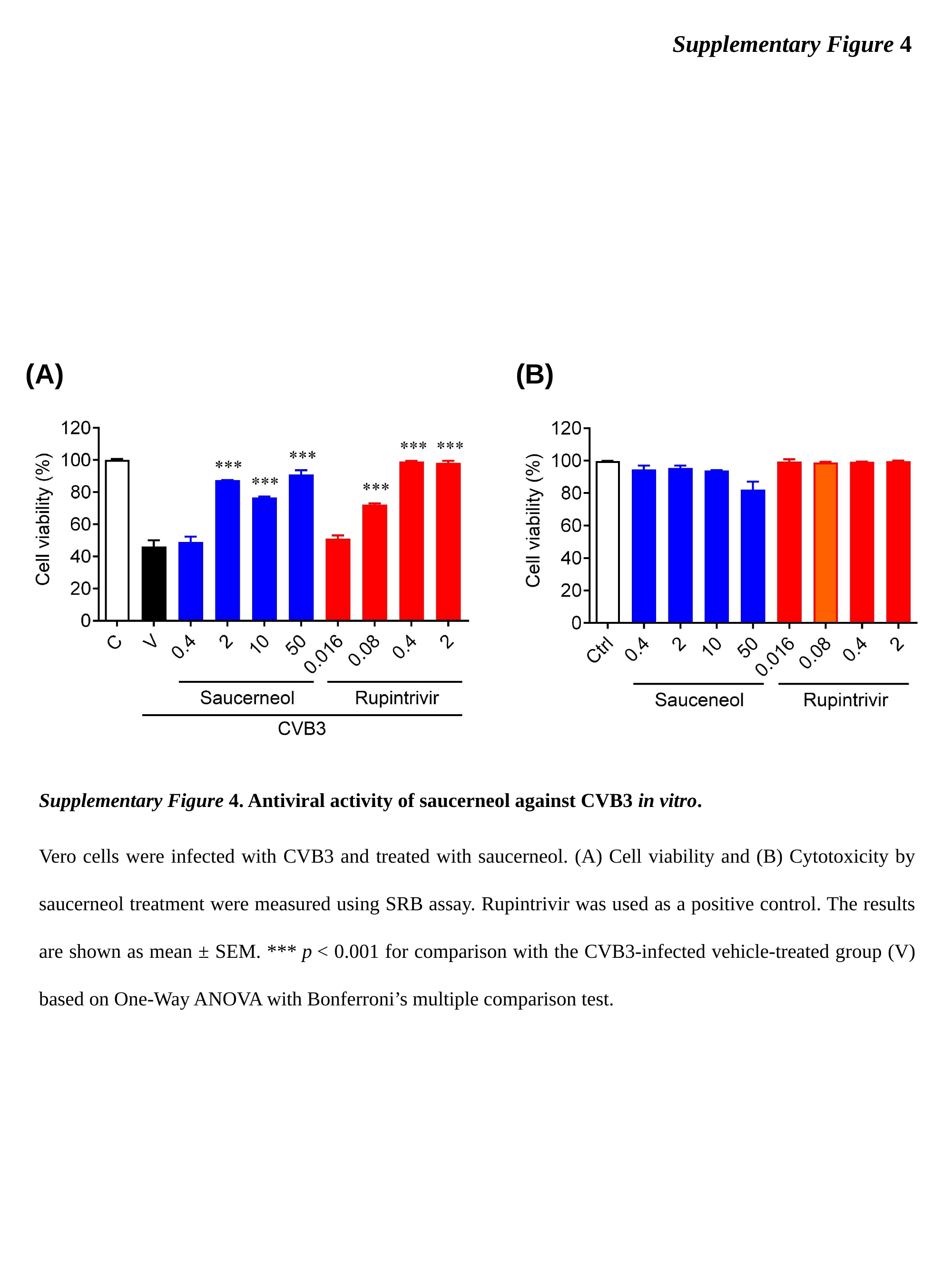

Supplementary Figure 4
(A)
(B)
Supplementary Figure 4. Antiviral activity of saucerneol against CVB3 in vitro.
Vero cells were infected with CVB3 and treated with saucerneol. (A) Cell viability and (B) Cytotoxicity by saucerneol treatment were measured using SRB assay. Rupintrivir was used as a positive control. The results are shown as mean ± SEM. *** p < 0.001 for comparison with the CVB3-infected vehicle-treated group (V) based on One-Way ANOVA with Bonferroni’s multiple comparison test.

## Slide 5
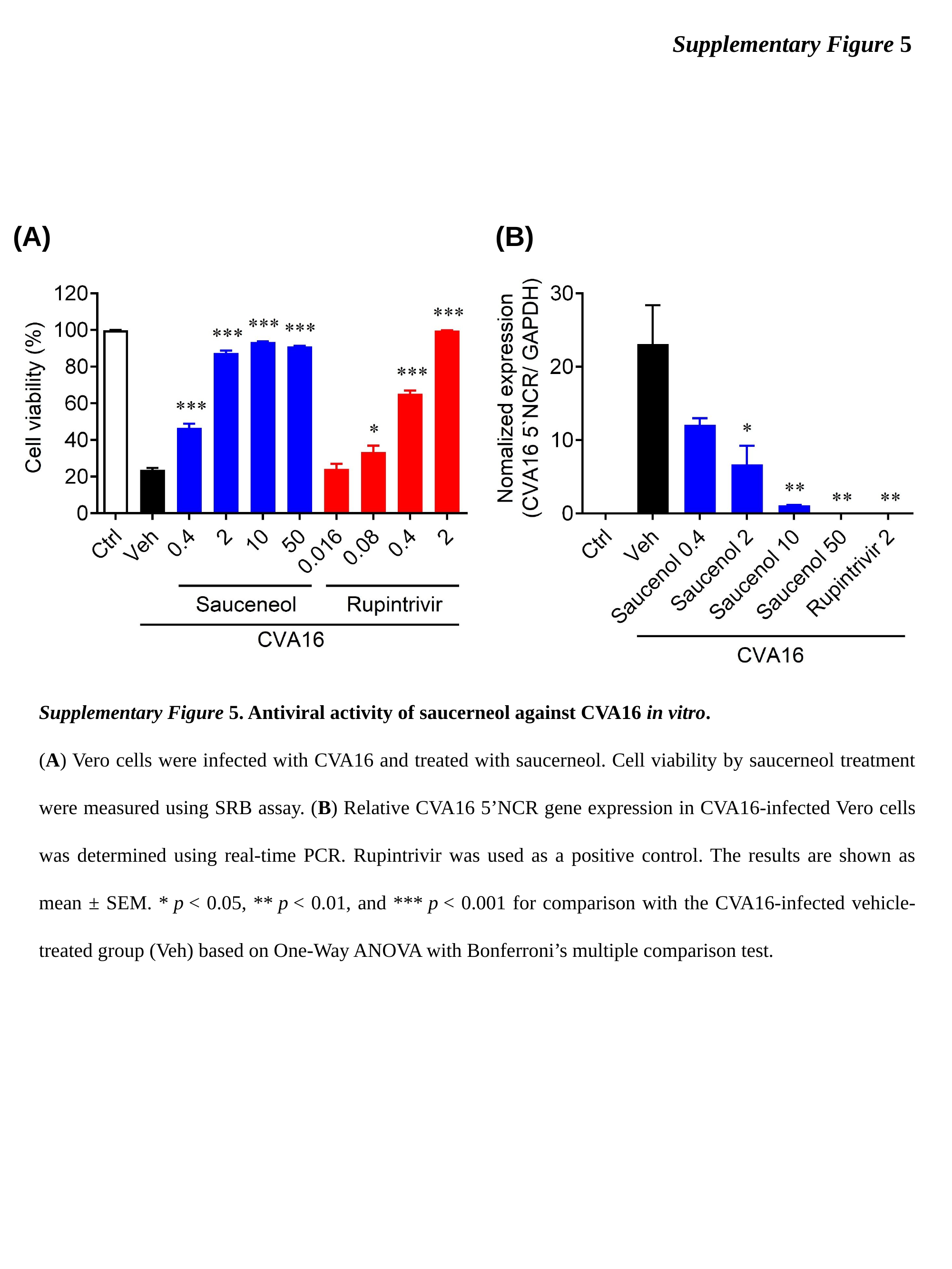

Supplementary Figure 5
(A)
(B)
Supplementary Figure 5. Antiviral activity of saucerneol against CVA16 in vitro.
(A) Vero cells were infected with CVA16 and treated with saucerneol. Cell viability by saucerneol treatment were measured using SRB assay. (B) Relative CVA16 5’NCR gene expression in CVA16-infected Vero cells was determined using real-time PCR. Rupintrivir was used as a positive control. The results are shown as mean ± SEM. * p < 0.05, ** p < 0.01, and *** p < 0.001 for comparison with the CVA16-infected vehicle-treated group (Veh) based on One-Way ANOVA with Bonferroni’s multiple comparison test.

## Slide 6
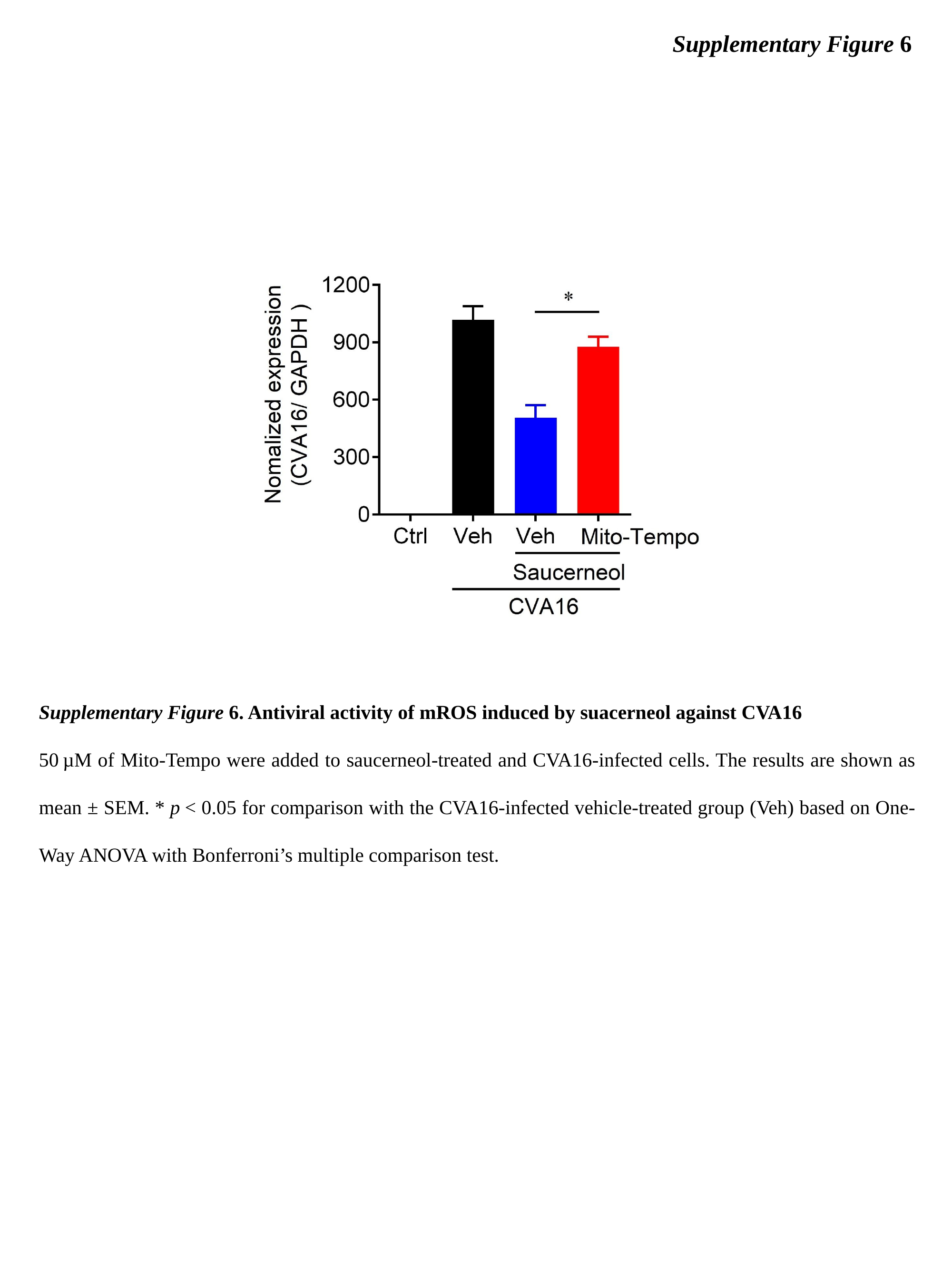

Supplementary Figure 6
Supplementary Figure 6. Antiviral activity of mROS induced by suacerneol against CVA16
50 µM of Mito-Tempo were added to saucerneol-treated and CVA16-infected cells. The results are shown as mean ± SEM. * p < 0.05 for comparison with the CVA16-infected vehicle-treated group (Veh) based on One-Way ANOVA with Bonferroni’s multiple comparison test.

## Slide 7
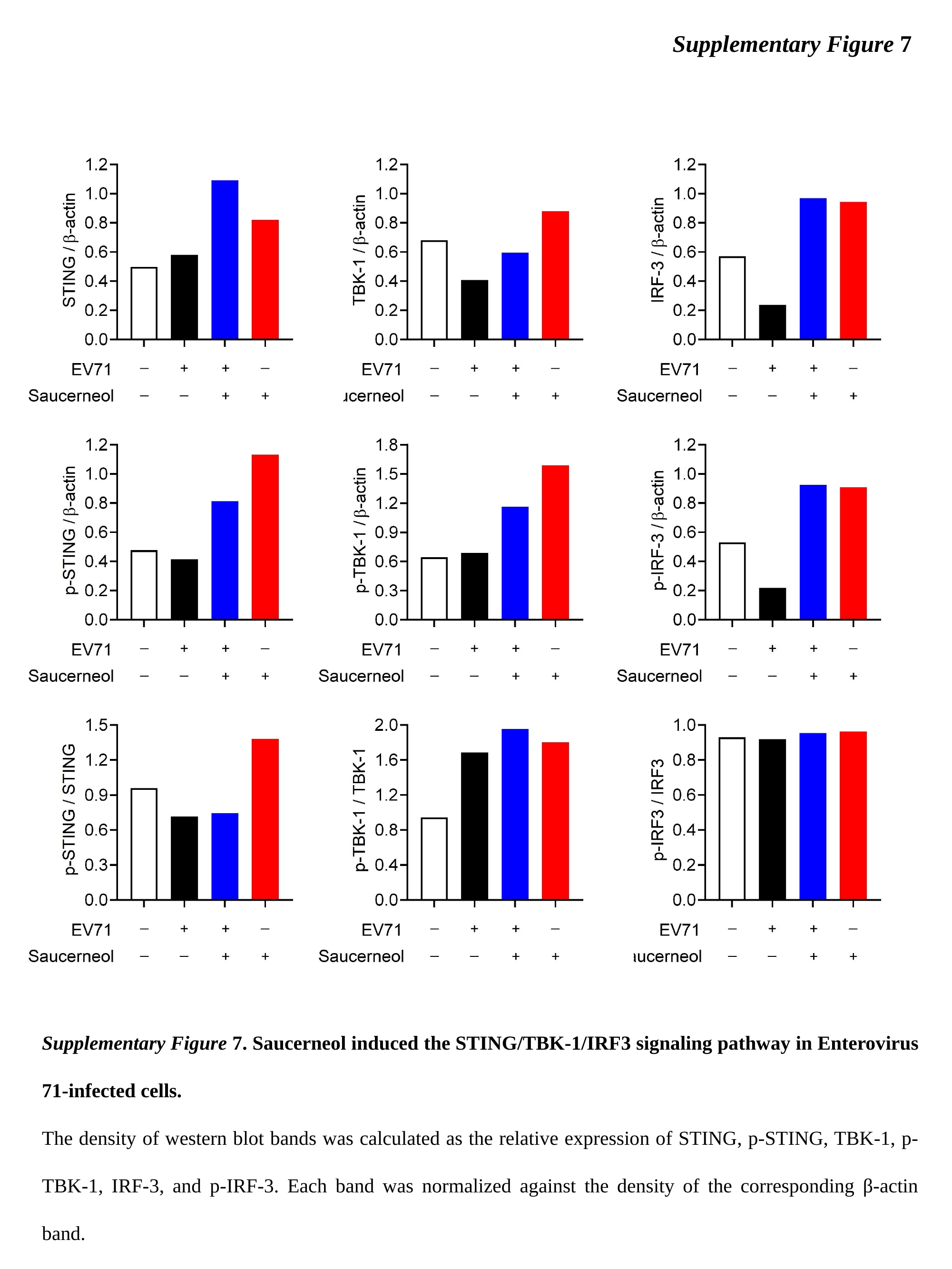

Supplementary Figure 7
Supplementary Figure 7. Saucerneol induced the STING/TBK-1/IRF3 signaling pathway in Enterovirus 71-infected cells.
The density of western blot bands was calculated as the relative expression of STING, p-STING, TBK-1, p-TBK-1, IRF-3, and p-IRF-3. Each band was normalized against the density of the corresponding β-actin band.

## Slide 8
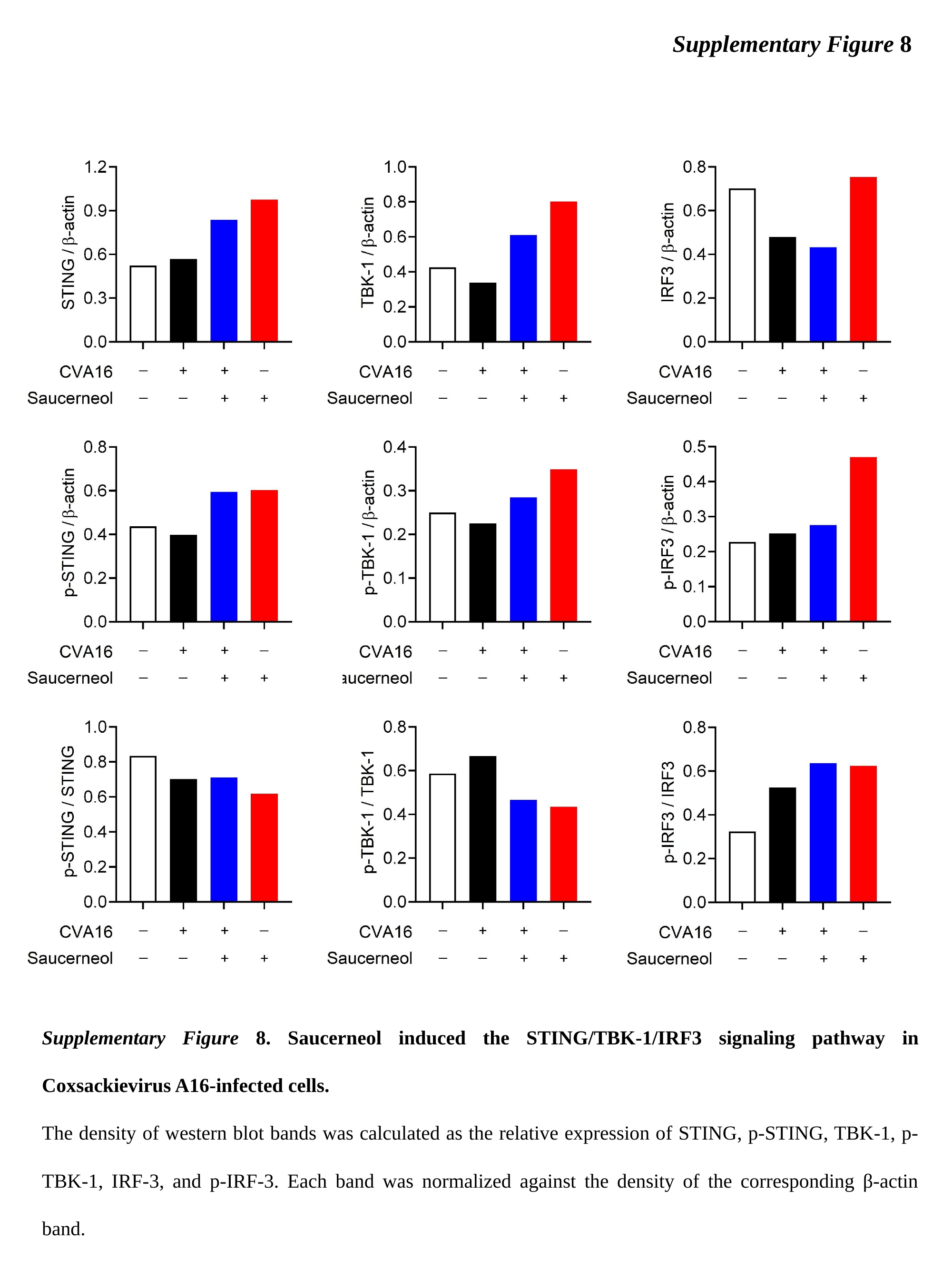

Supplementary Figure 8
Supplementary Figure 8. Saucerneol induced the STING/TBK-1/IRF3 signaling pathway in Coxsackievirus A16-infected cells.
The density of western blot bands was calculated as the relative expression of STING, p-STING, TBK-1, p-TBK-1, IRF-3, and p-IRF-3. Each band was normalized against the density of the corresponding β-actin band.
